# Differences in Stress Perception of Medical Students Depending on In-Person Communication and Online Communication during the COVID−19 Pandemic: A Japanese Cross-Sectional Survey

**DOI:** 10.3390/ijerph20021579

**Published:** 2023-01-15

**Authors:** Kazuki Tokumasu, Yoshito Nishimura, Yoko Sakamoto, Mikako Obika, Hitomi Kataoka, Fumio Otsuka

**Affiliations:** 1 Department of General Medicine, Okayama University Graduate School of Medicine, Dentistry and Pharmaceutical Sciences, Okayama 700-8558, Japan; 2 Department of Medicine, John A. Burns School of Medicine, University of Hawai’i, Honolulu, HI 96813, USA; 3 Center for Innovative Clinical Medicine, Okayama University Hospital, Okayama 700-8558, Japan; 4 Center for Diversity and Inclusion, Okayama University Hospital, Okayama 700-8558, Japan

**Keywords:** COVID-19, medical student, medical education, in-person communication, online communication, stress perception

## Abstract

Background: Excessive psychological stress in medical students affects their mental health and causes problems such as burnout and depression. Furthermore, changes in the learning environment to online learning due to the COVID-19 pandemic have had a psychological effect on medical students. However, the relationships between medical students’ perceived stress and different methods of communication, including in-person and online communication, remain unclear. The purpose of this study was to investigate the differences in stress perception of medical students depending on in-person communication and online communication during the COVID-19 pandemic. Methods: This study was a cross-sectional study conducted from September to October in 2020. All of the students of Okayama University School of Medicine were asked to participate in a questionnaire survey. The explanatory variables were the frequency and length of communications with others (by in-person or online communication), empathy, and lifestyle. The main outcome measure was perceived stress. Subgroup analysis was conducted for students who preferred to be by themselves and students who preferred to study together and interact with other people. Univariate analysis and multivariate multiple regression analysis were conducted. Gender and grade, which have been shown to be associated with stress in previous studies, were used as covariates for multiple regression analysis. Results: Valid responses to the questionnaire survey were received from 211 (29.4%) of the 717 students. There was no significant association between perceived stress and online communication, but the number of people with which students had in-person communication (1–2 people compared to 0 as a control, regression coefficient [B] = −4.4, 95% confidence interval [CI]; −7.8, −1.1, more than 10 people, B = −12, 95% CI: −18, −5.8) and the length of communication (more than 120 min, B = −4.5, 95% CI: −8.1, −0.92) were associated with a reduction in perceived stress. In subgroup analysis, the number of people with in-person communication and the length of communication had significant associations with stress reduction even in the group of students who had a preference for being by themselves. Conclusion: In-person communications rather than online communications were associated with a lower level of perceived stress. In subgroup analysis, this trend was statistically significant in the group of students who had a preference for being by themselves.

## 1. Background

In 2020, the global pandemic of COVID-19 had unprecedented medical and social consequences for all healthcare workers. Various factors during the pandemic including lack of resources, critical decision-making, patient briefing, information gathering, prevention, and pre-hospital response caused mental health issues for healthcare workers [1].

It was shown that medical students had significantly higher rates of psychological distress, mood disorders, and anxiety disorders than those of other university students [2,3]. The reasons for the higher rates in medical students were financial burden, sleep deprivation, overwhelming workload, and lack of free time [3]. Important personal challenges such as living alone for the first time and finding a new social role in one’s peer group were also reported to be reasons [4]. A feeling of guilt if they did not spend their limited spare time on learning and feelings of social isolation, especially during exam phases, were also reported as possible reasons [4]. During the COVID-19 pandemic, students have been forced to transfer to online-based learning and their in-person communication has been restricted. The learning environment has changed and the change in the learning environment has increased the psychological burden of students [2,5,6]. 

As in other countries [7], medical students in Japan have experienced psychiatric stress due to changes in the educational environment [8,9]. In a questionnaire survey conducted on medical students regarding students’ subjective psychological distress, it was found that students who expressed concerns about the rapid shift toward online education and anxiety about basic life security were more likely to be depressed and anxious after the surge in COVID-19 cases in Japan (April 2020) [9]. It was shown in that survey that 15.9% of the students had moderate-to-severe depression with a score of more than 10 in the Patient Health Questionnaire [9]. It was also shown in the survey that 29.8% of the students had concerns about the shift toward online education and the reasons were “online education may not be as effective as on-site education (65.2%)”, “fear of sudden change in the curriculum (52.5%)” and “less clinical exposure (65.2%) [9]. Many of the students who participated in that survey also had concerns about future career disruption, attenuated relationships with medical teachers, and disruption of ongoing extracurricular activities [9]. 

There have been several studies on perceived stress in health profession students. It has been shown that high levels of perceived stress strongly affect mental health and have unfavorable effects on academic performance [10,11]. It was also shown that medical students’ perceived stress was correlated with academic burnout in the environment of online learning during the COVID-19 pandemic [12]. Perceived stress is defined as a condition or feeling experienced when a person perceives that the demands exceed the personal and coping resources the individual can mobilize [13]. Facing the changes in the learning environment and living styles during the COVID-19 pandemic, the perceived stress in medical students could therefore be understood as an imbalance between their living and online learning needs during the pandemic [9,14]. It is important to identify and address the factors that have led to stress in medical students during the COVID-19 pandemic. Gender, grade, psychosocial stressors, health-related stressors, specific online learning behavior (persistence, attitude and flexibility), and the online learning environment (teaching, social and cognitive presence) were shown to be predictors of perceived stress in medical students [15]. Daily online learning time was shown to be associated with medical students’ perceived stress [16]. It was also shown that age, gender, watching news, worrying about the risk of infection, and the imposed curfew affected perceived stress in nursing students [17].

Some studies have revealed factors that are related to perceived stress in medical students [9,14,15,16,17]. However, the relationships between medical students’ perceived stress and different methods of communications, including in-person and online communication, remain unclear. The purpose of this study was to investigate the differences in stress perception of medical students depending on in-person communication and online communication during the COVID-19 pandemic.

### Literature Review about Mental Health Problems of Healthcare Workers during the COVID-19 Pandemic

The increase in the workload and stress experienced by healthcare workers have worsened mental health issues such as anxiety, burnout, and depression during the COVID-19 pandemic [18,19,20]. The reported prevalence of healthcare workers’ mental issues were 29.8% for stress, 24.1% for anxiety, and 13.5% for depression [21]. Another study conducted in Japan showed that the prevalence of burnout, depression, and job-related stress were 27%, 43%, and 62%, respectively [22]. A systematic review and meta-analysis focusing on the psychological impact of the COVID-19 pandemic on healthcare workers revealed a pooled prevalence of anxiety of 37% and a pooled prevalence of depression of 36% [23]. Several risk factors were shown to be associated with mental health issues during the COVID-19 pandemic [24,25]. Healthcare workers who were exposed to SARS-CoV-2-infected patients in emergency wards, wards for infectious diseases, and intensive care units were at a higher risk of showing symptoms of anxiety, depression, and sleep disorders than healthcare workers working in other wards [25]. Having a colleague who died was associated with perceived stress and symptoms of depression [24]. A large proportion of frontline healthcare workers suffered from anxiety and depression since frontline healthcare professionals treating patients with COVID-19 had a high risk of infection because of their frequent close contact with patients and long working hours [23]. These healthcare workers were also exposed to emotionally challenging interactions with sick and critically ill patients and they tended to pay more attention to their own health and their families’ health [23].

## 2. Methods

### 2.1. Study Design, Setting, and Participants

We performed a cross-sectional study using an anonymous, self-administered voluntary web-based survey. The participants were medical students in all years of study at the Okayama University of Medicine. We invited all of the students who belonged to the university as of 1 April 2020 (the first day of the academic year in Japan) to participate in the survey. Okayama is a regional city in western Japan and Okayama University belongs to the National University Corporation. At the time of the survey, first- to fourth-year medical students were in a classroom-based learning environment and were preparing assignments in online or on-demand classes. Fifth-year medical students were in clinical clerkship and sixth-year medical students were taking their graduation examinations.

The survey was administered by Qualtrics (Qualtrics International Inc., Provo, UT, USA), a web-based survey platform. We provided survey instructions and instruments in Japanese. We distributed survey links to the students using students’ official mailing lists. All participants were invited to complete the survey within four weeks (28 September 2020 and reminded on 9 October and 23 October in Japan Standard Time). No financial incentives were provided for their participation in the survey. In the survey, we included entries on demographics (age, gender, grade, living environment, purpose of SNS, preference for being by themselves, and the Jefferson Scale of Empathy), learning activities (e.g., hours of self-learning at home, hours of taking lectures) as well as communication characteristics (e.g., frequency of communication, duration of communication) and perceived stress scale.

### 2.2. Measurements

Questions regarding explanatory variables included personal, lifestyle, learning environment and communication questions (details of the questionnaire are shown in the File S1). Personal and lifestyle questions included questions on grade, gender, age, living situation (with whom medical students live) and preference for being by themselves. Learning environment questions included questions on average hours spent at home, online lecture time and self-study time on weekdays and weekends. Communication questions included questions on the current amount of communication compared with that before the COVID-19 pandemic, how many people medical students have conversations with either in-person or online in one week, the length of time spent interacting with these people per communication (in-person or online) and purpose of using social networking services.

### 2.3. Perceived Stress Scale

We assessed the presence of psychiatric stress using the Japanese version of the Perceived Stress Scale (PSS) [26,27]. The PSS, developed by Cohen et al. [13], was used in this study to assess students’ stress levels. The PSS consists of 14 items and includes questions such as ‘In the last month, how often have you been upset because of something that happened unexpectedly?’ and ‘In the last month, how often have you felt that you were unable to control the important things in your life?’ Responses are coded for scoring as never = 0, almost never = 1, sometimes = 2, fairly often = 3 and very often = 4. Possible total scores range from 0 to 56, with higher scores indicating higher levels of negative cognitive stress appraisal. All of the 14 items in the Japanese version of the scale are highly intercorrelated (Cronbach’s alpha = 0.76) [27].

### 2.4. Statistical Analysis

First, descriptive statistics were calculated for subject attributes and explanatory factors. Second, univariate analysis and multiple regression linear analysis were conducted with the PSS as the outcome variable and each of the communication material variables as the exposure variable. Gender and grade, which have been shown to be associated with stress in previous studies [15,17], were used as covariates.

Subgroup analysis was conducted for students who prefer to be by themselves and students who prefer to study together with and interact with other people. The significance level was set at α = 0.05. We reported point estimates of regression coefficients and 95% confidence intervals. All statistical analyses were performed using Stata/SE 17.0 (Stata Corp, College Station, TX, USA). A *p*-value of less than 0.05 was considered statistically significant. 

### 2.5. Informed Consent and Reporting Checklist

The questionnaire included explanations on participation, and participants were included in the study after checking whether informed consent had been obtained from them. Furthermore, opportunities to refuse after participating in the study were provided on the study site’s website. While writing this paper, we used the STROBE cross-sectional checklist [28]. 

## 3. Results

There were 717 medical students in Okayama University at the time of the survey. Of all of the students, 286 responded and 75 had missing data. Therefore, 211 students were finally included in the analysis. The participants included 75 females (35.6%) and the median age of the participants was 22.5 years.

Basic demographics of the participants such as grade, gender, and time spent at home and class time are summarized in Table 1 and Table 2. During the COVID-19 pandemic, more than 50% of the students lived alone without any relatives nearby. The students stayed at home for more than 20 h each day on both weekdays and weekends. The total amount of subjective communication was about 50% smaller than that in the pre-COVID-19 pandemic period (Table 2). A total of 130 students (61.6%) answered that they preferred to be by themselves. The average PSS score was 30.3 (males: 29.1, females: 32.1), indicating that the medical students had a moderate level of perceived stress, predominantly in females (regression coefficient [B] = 3.3, 95% confidence interval [CI]: 0.6, 5.9). The results of multiple linear regression analyses showed that a higher PSS score in Japanese medical students was associated with individual factors (do not prefer to be by themselves, B = 3.3, 95% CI: 0.6, 6.1) and longer hours staying at home (total hours through weekdays and weekends, B = 0.2, 95% CI: 0.004, 0.4) (Table 3).

There was no significant association between perceived stress and online communication, but the number of people with which students had in-person communication (1–2 people compared to 0 as a control, B = −4.4, 95% CI: −7.8, −1.1; more than 10 people, B = −12, 95% CI: −18, −5.8) and the length of in-person communication (more than 120 min, B = −4.5, 95% CI: −8.1, −0.92) were significantly associated with perceived stress reduction (Table 3). In subgroup analysis, the number of people with in-person communication and the length of in-person communication had significant associations with stress reduction even in the group of students who preferred to be by themselves (Table 4).

## 4. Discussion

In this study, the PSS and its impact on living styles, self-study time, class time, and modes of in-person and online communication were investigated in Japanese medical students. As was found in previous studies [15,17], the stress scale was higher in female students in this study. Female medical students are more likely than male medical students to experience different expectations, pressures, obstacles, and harassment in the process of developing the capacity to gain scientific excellence and gender equality-related management positions [29]. As one of the possible reasons for this, a previous study revealed that the higher level of perceived stress in females was due to the differences in neuroendocrine and hypothalamic–pituitary–adrenal axis reactivity [30]. Although some studies have shown higher stress indices in younger grades [15], the difference in stress indices by grade in this study was not statistically significant.

The high percentage of medical students living alone (69.2–73.9%) in this study was thought to be due to the fact that the medical school was a national university located in a rural area. A previous survey showed that the percentage of students living alone was higher in rural areas and for national universities than in urban areas and for private universities in Japan. In that survey, the percentage of university students who were living alone was 69.5% [31]. The high percentage of students living alone might be the reason for a high proportion of students not having in-person communication with anyone because of the COVID-19 pandemic and campus lockdown [9,15].

In a survey of the lives of students of national universities before the COVID-19 pandemic, the students reported that the items accounting for more than 1.6 h/day in a typical day during the school term were club activities (13.6% of the students), part-time/regular work (50.3%), and entertainment/fellowship (41%) [31]. Given that the average time spent at home in this study was 20.9–21.3 hours/day, it can be inferred that the COVID-19 pandemic had led to a decline in external activities. In a study conducted in the same population as that in the present study, 9.3% of the medical students stopped working due to the COVID-19 pandemic during the period of the COVID-19 lockdown [9]. Indeed, physical activity was reduced by the COVID-19 pandemic worldwide [32].

In this study, no significant association was found between perceived stress and online communication, but the number of people with which students had in-person communication and the length of in-person communication were significantly associated with a reduction in perceived stress. In general, positive interpersonal communication reduces stress perception [33] and a previous study in which conversations and perceived stress among college students were surveyed revealed a negative correlation between a higher frequency and longer conversations with perceived stress [34].

Online communication did not have a significant association with medical students’ perceived stress reduction in this study. Indeed, a number of previous studies have shown negative effects of online communication on psychological well-being [35,36,37]. A 2-year prospective study showed that frequent online communications may directly reduce the psychosocial well-being of adolescents [36]. In that study, it was found that teenagers who spent more time online experienced a greater decline in social and psychological well-being during the first year of access to the Internet. Teenagers who were lonely and depressed were more attracted to the Internet, but the results of the study suggested that using the Internet was related to a decrease in social well-being. A possible explanation for the results is that adolescents’ heavy usage of the Internet for online communication led them to forsake critical relations with local friends and family for weak relations with strangers. It is also possible that medical students in this study had difficulty establishing relationships using online communication.

In the subgroup analysis, the number of people with whom students had in-person communication and the length of in-person communication had significant associations with stress reduction even in the group of students that had a preference for being by themselves. It is possible that people who originally did not prefer being by themselves had a habit of studying together and interacting with other people. The COVID-19 pandemic might have forced almost all medical students to experience loneliness, and those who originally preferred being by themselves may not have known how to cope with loneliness. Furthermore, Japanese people have been shown to be more interdependent than Westerners [38], and it may have been difficult for medical students to form a community in the situation of campus lockdown. It is possible that in-person communication may be better than online communication for reducing perceived stress.

### Limitations and Further Research

The present study has several limitations. First, since this study was an exploratory cross-sectional study, causal effects were not identified. Therefore, the interpretation of the results for generalization is limited. Second, the full response rate for the web-based questionnaire was not high (29.4%). The reasons for this might be that some medical students had difficulties with Internet access and that some students had a low frequency of email checks. Third, the analyses we conducted should be interpreted as exploratory because of the potential risk for a type I error due to multiple testing.

The COVID-19 pandemic has changed the learning environment for medical students, with classes going online and learning communities changing. For further research, a detailed investigation of the relationship between students’ perceived stress and psychological disease is warranted. Additionally, a previous study showed a relationship between psychiatric response to stress and coping strategies [39]. It is likely that medical students were stressed during the COVID-19 pandemic, and it is, therefore, important to determine how they coped with this stress.

## 5. Conclusions

Factors contributing to perceived stress among Japanese medical students during the COVID-19 pandemic were investigated in this study. In-person communications rather than online communications were associated with a lower level of perceived stress. In the subgroup analysis, this trend was statistically significant in the group of students who had a preference for being by themselves.

## Figures and Tables

**Table 1:** Participants’ Characteristics.

Characteristic	Value	% (SD)
Age in years ^a^		
Mean	22.5	(3.26)
Gender, No.		
Female	75	35.6
Male	134	63.5
Not confirmed	2	0.1
Grade, No.		
First	40	19.0
Second	32	15.2
Third	31	14.7
Forth	31	14.7
Fifth	42	19.9
Sixth	35	16.6
Total number of participants	211	

Abbreviations: CI, confidence interval; SD, standard deviation. ^a^ A total of 2 of the 211 respondents did not specify their age.

**Table 2:** Demographic Characteristics of the Study Participants.

Characteristic	Value	% (SD)
Living status, No. At the time the survey was conducted (October 2020)		
Living alone without any relatives nearby	118	55.9
Living alone with relatives nearby	38	18.0
Living with someone	55	26.1
Living status, No. Most stressful time		
Living alone without any relatives nearby	111	52.6
Living alone with relatives nearby	35	16.6
Living with someone	65	30.1
Purpose of using SNS, No.		
Communication	114	54.0
Gathering information	97	46.0
Preference to be by themselves, No.		
Yes	130	61.6
No	81	38.4
Staying at home, hours		
Weekdays, mean	20.9	(3.9)
Weekends, mean	21.3	(3.7)
Taking online lectures, hours		
Weekdays, mean	3.3	(3.1)
Self-learning at home, hous		
Weekdays, mean	3.2	(3.0)
Weekends, mean	3.9	(4.2)
Communication		
Change in the amount of communication compared to the pre-COVID-19 pandemic period, mean %	51.5	(52.1)
Number of people with in-person communication within one week, No.		
0	47	22.3
1–2 persons	88	41.7
3–10 persons	64	30.3
>10 persons	12	5.7
Number of people with online communication within one week, No.		
0	13	6.2
1–2 persons	56	26.5
3–10 persons	124	58.8
>10 persons	18	8.5
Length of in-person communication per communication, No.		
<15 min	71	33.7
15–30 min	35	16.6
30–120 min	56	26.5
>120 min	49	23.2
Length of online communication per communication, No.		
<15 min	54	25.6
15–30 min	38	18.0
30–120 min	83	39.3
>120 min	36	17.1
Total number of participants	211	

Abbreviations: CI, confidence interval; SD, standard deviation; SNS, social networking system.

**Table 3:** Results of Multiple Linear Regression Analyses of Explanatory Variables Associated with Perceived Stress Scale Japanese in Medical Students.

		Crude Model	Adjusted Model (Grades and Gender)
Explanatory Variable		Coef (95% CI ^b^)	Coef (95% CI ^b^)
Gender (male vs. female)		3.3 * (0.6, 5.9)	
Grade		−0.2 (−0.9, 0.6)	
SNS purpose (communication vs. gathering information?)		−1.1 (−3.8, 1.6)	−1.0 (−3.7, 1.7)
Preference to be by themselves (yes vs. no)		3.4 * (0.7, 6.2)	3.3 * (0.6, 6.1)
Jefferson Scale of Empathy (student version)		−0.007 (−1.0, 0.09)	−0.1 (−0.1, 0.8)
At the time the survey was conducted (September–October 2020)			
Living alone without any relatives nearby		ref	ref
Living alone with relatives nearby		−1.4 (−5.1, 2.2)	−1.3 (−4.9, 2.3)
Living with someone		0.51 (−2.7, 3.7)	0.38 (−2.8, 3.6)
Most stressful time			
Living alone without any relatives nearby		ref	ref
Living alone with relatives nearby		−1.3 (−5.1, 2.5)	−0.8 (−4.6, 3.0)
Living with someone		0.6 (−2.5, 3.7)	0.5 (−2.6, 3.5)
Staying at home; weekdays		0.3 (−0.0004, 0.7)	0.3 (−0.02, 0.7)
Staying at home; weekends		0.4 (−0.02, 0.7)	0.3 (−0.02, 0.7)
Staying at home; total		0.2 * (0.01, 0.4)	0.2 * (0.004, 0.4)
Taking online lectures		0.1 (−0.3, 0.6)	−0.06 (−0.6, 0.5)
Self-learning at home; weekdays		0.1 (−0.3, 0.6)	−0.01 (−0.5, 0.5)
Self-learning at home; weekends		0.09 (−0.2, 0.4)	−0.02 (−0.4, 0.3)
Self-learning at home; total		0.06 (−0.1, 0.3)	−0.01 (−0.2, 0.2)
Amount of communication		−0.03 * (−0.06, −0.006)	−0.04 * (−0.06, 0.01)
Number of people (in-person)	0	ref	ref
	1–2 persons	−4.1 * (−7.5, −0.6)	−4.4 * (−7.8, −1.1)
	3–10 persons	−2.7 (−6.4, 0.9)	−3.3 (−6.9, 0.3)
	>10 persons	−12 ** (−18, −5.6)	−12 ** (−18,−5.8)
Number of people (online)	0	ref	ref
	1–2 persons	−3.8 (−9.8, 2.3)	−2.4 (−8.5, 3.7)
	3–10 persons	−3.9 (−9.6, 1.8)	−3.1 (−8.8, 2.7)
	>10 persons	−3.3 (−10, 3.9)	−3.1 (−10, 4.1)
Length (in-person)	<15 min	ref	ref
	15–30 min	−3.5 (−7.5, 0.6)	−3.2 (−7.3, 0.8)
	30–120 min	−1.6 (−5.1, 1.8)	−1.9 (−5.3, 1.6)
	>120 min	−4.5 * (−8.2, −0.9)	−4.5 * (−8.1, 0.9)
Length (online)	<15 min	ref	ref
	15–30 min	−1.1 (−5.2, 3.1)	−1.6 (−5.8, 2.5)
	30–120 min	−1.2 (−4.6, 2.2)	−1.9 (−5.4, 1.6)
	>120 min	−3.3 (−7.4, 0.9)	−3.9 (−8.2, 0.4)

* *p*-value < 0.05, ** *p*-value < 0.01. ^b^ Multiple linear regression analysis was performed for each dependent variable with grades and gender as covariates.

**Table 4:** Results of Univariate Regression Analyses of Communication Factors Associated with Perceived Stress Scale in Japanese Medical Students. (Subgroup analysis of students who prefer to be by themselves and students who prefer to be with other people.)

		Group 1		Group 2	
Explanatory Variable		Coef	95% CI ^b^	Coef	95% CI ^b^
Amount of Communication		−0.04 *	−0.07, −0.005	−0.03	−0.07, 0.009
Number of people (in-person)	0	ref	ref	ref	ref
	1–2 persons	−4.9 *	−9.6, −0.3	−1.9	−6.8, 3.0
	3–10 persons	−4.5	−9.4, 0.4	−0.3	−5.6, 4.9
	>10 persons	−12 **	−19, −4.0	−12	−23, 0.2
Number of people (online)	0	ref	ref	ref	ref
	1–2 persons	−0.89	−8.7, 7.0	−4.5	−14, 4.6
	3–10 persons	0.50	−7.1, 8.1	−8.4	−17, 0.08
	>10 persons	−3.0	−13, 6.7	−3.8	−14, 6.7
Length (in-person)	<15 min	ref	ref	ref	ref
	15–30 min	−2.1	−7.3, 3.0	−1.4	−8.2, 5.3
	30–120 min	−2.0	−6.5, 2.6	−0.06	−5.1, 4.9
	>120 min	−7.5 **	−12, −2.8	1.1	−4.1, 6.4
Length (online)	<15 min	ref	ref	ref	ref
	15–30 min	−1.0	−6.3, 4.2	−1.5	−8.2, 5.1
	30–120 min	−0.60	−5.1, 3.9	−3.0	−8.7, 2.7
	>120 min	−4.2	−10, 1.6	−3.6	−10, 3.0

* *p*-value < 0.05, ** *p*-value < 0.01. Group 1: Medical students who had a preference for being by themselves. Group 2: Medical students who had a preference for studying together with and interacting with other people. ^b^ Multiple linear regression analysis was performed for each dependent variable with grades and gender as covariates.

## Data Availability

The datasets generated and analyzed during the current study are available from the corresponding author on reasonable request.

## Supplementary Materials

The following supporting information can be downloaded at: https://www.mdpi.com/article/10.3390/ijerph20021579/s1, File S1: Questionnaires.

## Abbreviations

COVID-19	Coronavirus disease 2019
PSS	perceived stress scale
SNS	social networking service
