# Supplementary material for: Differences in Stress Perception of Medical Students Depending on In-Person Communication and Online Communication during the COVID−19 Pandemic: A Japanese Cross-Sectional Survey
Source: Int J Environ Res Public Health. 2023 Jan 15;20(2):1579. doi: 10.3390/ijerph20021579 (PMC9865011; doi:10.3390/ijerph20021579)
Supplement: Supplementary file 1 [file ijerph-20-01579-s001.zip › ijerph-2147666-supplementary.pdf]

Supplement data: Questionaries

1. Recall the time when the new coronavirus outbreak was most stressful for you.

a. What was the date? Year/month/day

b. In what department was your clinical training and what lectures were being offered? Or were you on holiday? (free text)

2. Average hours you spent at home, online lectures time and self-study time on weekdays and weekends.

a. Weekdays: average hours spent on online lectures per day 0-24hr

b. Weekdays: average hours of self-study per person per day 0-24hr

c. Weekdays: average hours spent at home per day 0-24hr

d. Weekends: average hours spent studying alone per day 0-24hr

e. Weekend: average hours spent at home per day 0-24hr

3. Communication questions.

a. Throughout the weekdays and weekends, how many people did you have in-person conversations within total in a week? 0 people/1-2 people/3-5 people/6-10 people/11 or more people

b. On average, how long did you spend interacting with these people per communication? (in-person)
Less than 15 minutes/15~30 minutes/30-60 minutes/60-120 minutes/more than 120 minutes

c. How many people did you have online conversations with (e.g., by phone, video call, social networking message) in total in a week? 0 people/1-2 people/3-5 people/6-10 people/11 or more people

d. On average, how long did you communicate with these people per communication? (Online)
Less than 15 minutes/15~30 minutes/30-60 minutes/60-120 minutes/more than 120 minutes

e. If you used social networking services (SNS), for which of the following main purposes did you use them?
Gathering information/Communicating with other people

f. What was the amount of communication with people compared to that before the COVID-19 pandemic?
: 0------50-----100-----150-----200-----250-----300 (%)

4. Personal questions.

a. What is your grade in medical school? 1/2/3/4/5/6

a. Gender Male/Female/Other

b. Age

c. We would like to ask you about your current living situation.
Living alone and no relatives or family nearby/Living alone but with relatives or family nearby/Living with a partner/Living with a roommate/Living with a family member

d. How was your life during the stressful period of the outbreak of the new coronavirus infection that you answered above?
Living alone and no relatives or family nearby/Living alone but with relatives or family nearby/Living with a partner/Living with a roommate/Living with a family member

e. Do you prefer to be by yourself?
Prefer/Not prefer/Neither

※The students who had conversations with 3-5 and 6-10 people (both in-person and online) per week were analyzed as a combined group due to their approximation as a group, although they were categorized in the questionnaire.

※Students with durations of communication per conversation (both in-person and online) of 30-60 minutes and 60-120 minutes were categorized in the questionnaire but were analyzed as a combined group due to their approximation as a group.

※The students who answered, "Not prefer" and "Neither" were analyzed as a combined group. This was because the aim of this study was to determine whether medical students preferred to be by themselves or not as a stress factor.
